# Analysis of Proprioceptive Sensory Innervation of the Mouse Soleus: A Whole-Mount Muscle Approach

**DOI:** 10.1371/journal.pone.0170751

**Published:** 2017-01-25

**Authors:** Martha J. Sonner, Marie C. Walters, David R. Ladle

**Affiliations:** Department of Neuroscience, Cell Biology, and Physiology, Wright State University, Dayton, Ohio, United States of America; University of Sydney, AUSTRALIA

## Abstract

Muscle proprioceptive afferents provide feedback critical for successful execution of motor tasks via specialized mechanoreceptors housed within skeletal muscles: muscle spindles, supplied by group Ia and group II afferents, and Golgi tendon organs, supplied by group Ib afferents. The morphology of these proprioceptors and their associated afferents has been studied extensively in the cat soleus, and to a lesser degree, in the rat; however, quantitative analyses of proprioceptive innervation in the mouse soleus are comparatively limited. The present study employed genetically-encoded fluorescent reporting systems to label and analyze muscle spindles, Golgi tendon organs, and the proprioceptive sensory neuron subpopulations supplying them within the intact mouse soleus muscle using high magnification confocal microscopy. Total proprioceptive receptors numbered 11.3 ± 0.4 and 5.2 ± 0.2 for muscle spindles and Golgi tendon organs, respectively, and these receptor counts varied independently (*n* = 27 muscles). Analogous to findings in the rat, muscle spindles analyzed were most frequently supplied by two proprioceptive afferents, and in the majority of instances, both were classified as primary endings using established morphological criteria. Secondary endings were most frequently observed when spindle associated afferents totaled three or more. The mean diameter of primary and secondary afferent axons differed significantly, but the distributions overlap more than previously observed in cat and rat studies.

## Introduction

Continual monitoring of alterations in muscle length, corresponding joint angle changes, and forces produced during muscle contraction are critical for execution of motor tasks. Proprioceptive sensory neurons (PSNs) encode and relay this information to the central nervous system for interpretation and response via spinal circuits and ascending pathways into the brain [1,2]. Axons extending into the periphery from PSN cell bodies localized in the dorsal root ganglia (DRG), supply specialized sensory receptors located in skeletal muscle, known as muscle spindles (MS) and Golgi tendon organs (GTOs).

MS and GTOs are both encapsulated, stretch-activated sensory receptors found within skeletal muscles. As a consequence of their differing intramuscular location and architecture, however, the PSNs that innervate each receptor respond to distinct physical stimuli. For example, MS are located in the belly of the muscle, positioned in parallel with extrafusal muscle fibers causing them to respond to muscle stretch [3]. Conversely, GTOs are found at myotendinous junctions, and their arrangement in series with muscle fibers enables their sensitivity to muscle contraction [4]. Other morphological features are also distinguishing. GTOs are innervated by a single afferent (group Ib) which branches extensively and intercalates with collagen fibers inside the capsule at the point where muscle meets tendon. A typical GTO is associated with a small group of muscle fibers and not all muscle fibers feed into GTOs [4–6]. Based on ratio calculations performed in cat hindlimb experiments, it is thought that the relatively small number of GTOs populating a given muscle is enough to adequately track motor unit activity [7].

MS are structurally more complex and are generally comprised of three types of intrafusal muscle fibers (termed bag1, bag2, and chain fibers according to nuclear arrangement) surrounded by a capsule structure [3]. The MS is supplied by at least one Ia afferent, variable numbers of group II afferents, and gamma-motor neuron axons that control intrafusal muscle fiber contraction. The Ia afferents contact each type of intrafusal fiber and form primary endings that have a characteristic annulospiral morphology. Terminations of group II afferents are referred to as secondary endings, and are found predominantly on chain intrafusal fibers with either spiral-like or flower-spray morphology [8–11].

As a result of their distinct endings on the muscle spindle, group II afferents encode stretch-evoked stimuli differently than Ia spindle afferents. For example, Ia afferents display a supralinear action potential firing response during passive muscle stretch, together with a characteristic pause in firing when the muscle is shortened [12]. Firing rates of group II afferents, on the other hand, are linear with muscle length, whether the muscle is being passively stretched, held at a new length, or shortened [12–14]. As a result, group Ia afferents better encode stretch velocity or dynamic stretch, whereas group II afferents relay information regarding maintained or static stretch [15].

To date, knowledge of the anatomy and physiology of MS and GTO has come primarily from work in the cat, and secondarily from studies in rat [3,8,10,16]. There is limited anatomical data beyond receptor counts for rodents, and more particularly for the mouse, despite the opportunities afforded by transgenic mouse models to investigate both the form and function of these receptors [16,17]. In this study we exploited mouse genetic tools to analyze the structure and afferent supply of MS and GTOs in a whole-mount preparation of the soleus muscle, a muscle thoroughly examined in the cat literature. Using intact muscles, we were able to systematically classify Ib endings in GTOs and both primary and secondary proprioceptive endings in MS, revealing similarities and differences in proprioceptive innervation between these two important animal models.

## Materials and Methods

### Animals

All animal experimental procedures were approved by the Wright State University Institutional Animal Care and Use Committee. A Cre-dependent conditional tdTomato fluorescent reporter mouse line (*Rosa26*^*tdTom/+*^ termed here *R26-tdT*; JAX Stock 007908) was crossed with two Cre-driver lines to label either all peripheral sensory neurons (*Advillin-Cre* (*Adv-Cre*); [18]) or the subset of neurons innervating proprioceptive endings in muscle (*Parvalbumin-Cre* (*PV-Cre*); JAX Stock 008069; [19]). Both male (n = 13) and female (n = 12) mice from two postnatal stages were used in this study. *PV-Cre; R26-tdT* animals were used from postnatal day 3 to 7 (P3—P7). Analysis of MS and GTOs in older animals (P19—P20) was performed using *Adv-Cre; R26-tdT* mice.

### Whole-Mount Soleus Muscle Preparation

Animals were anesthetized on ice (up to P7) or by Euthasol injection (older than P7) and transcardially perfused with 5 mL of ice-cold oxygenated (95% O2; 5% CO2) artificial cerebrospinal fluid (ACSF) containing: 127 mM NaCl, 1.9 mM KCl, 1.2 mM KH2PO4, 1 mM MgSO4·7H2O, 26 mM NaHCO3, 16.9 mM D(+)-glucose monohydrate, and 2 mM CaCl2. The animals were then decapitated and soleus muscle dissections were performed with the animal preparation submerged in a recirculating bath of cold, oxygenated ACSF. Following excision, soleus muscles to be analyzed for proprioceptive afferent quantification and diameter measurement were carefully pressed between two glass slides (25 x 75 mm, 1.0 mm thick), each prepared with a layer of filter paper and 40 μm cell strainer mesh, and then secured together with adhesive tape (method adapted from Vult von Steyern et al., 1999). This entire compression apparatus was immediately submerged in 40 mL of 4% PFA solution for 1 hour at 4°C. In this manner, muscle compression and fixation were accomplished simultaneously as the filter paper wicked the PFA in between the two slides, while the cell strainer mesh provided a PFA-accessible yet non-traumatic protective covering for the soleus muscle. After fixation, the compressed soleus muscle was rinsed three times with 1X PBS, mounted on a Superfrost Plus microscope slide with Vectashield medium (H-1000, Vector Laboratories, Burlingame, CA) and a glass coverslip in preparation for imaging and analysis.

Soleus muscles intended only for rapid muscle proprioceptive receptor quantification were mounted on a slide using Vectashield and a glass coverslip following transcardial perfusion and quick isolation in cold 1X PBS. No fixation was used in these preparations. Images of fresh dissected muscles were immediately acquired using an Olympus BX51 microscope (4x and 10x objectives) with SPOT RT Slider 2.3.0 color camera and SPOT Advanced software version 5.1 (Diagnostic Instruments, Inc.).

### Immunohistochemistry

Age P3 *PV-Cre; R26-tdT* and age P22 *Adv-Cre; R26-tdT* mice were anesthetized as described above and transcardially perfused with 10 mL of ice-cold 1X PBS followed by 10 mL of 4% paraformaldehyde (PFA) solution. The animals were then decapitated and all musculature superficial to the soleus muscle (hamstrings, lateral and medial gastrocnemius muscles) was removed from the hind limbs, and the branch of the tibial nerve supplying the soleus muscle was cut proximal to the point where the thin branch defasciculates. The entire lower hind limb, including exposed soleus muscle, was then submerged in 15 mL of 4% PFA for 2 hours at 4°C. After fixation, the lower hind limb was washed two times with 1X PBS and then equilibrated in sucrose solution (30% in PBS) overnight at 4°C for cryoprotection. The soleus muscle was then removed from the limb, embedded in tissue freezing medium and stored at -80°C until sectioning. Using a HM 550 cryostat, 20 μm-thick serial longitudinal sections were obtained and mounted on slides in preparation for immunohistochemistry. The soleus muscle sections were washed three times with 1X PBS and then incubated overnight at 4°C in an antibody solution (1X PBS with 1% bovine serum albumin and 0.3% Triton X-100) containing guinea pig anti-VGLUT1 polyclonal antibody diluted 1:10,000 (Chemicon AB5905, Lot LV1567574). Slides were then washed three times with 1X PBS and incubated for 45 minutes at 22°C in antibody solution containing Alexa Fluor 488 goat anti-guinea pig antibody diluted 1:1000 (Invitrogen A11073, Lot 455283). Following the secondary incubation, the slides were washed three times with 1X PBS. Vectashield mounting medium was then applied to the sections for fluorescence preservation prior to placement of a glass coverslip. High magnification images of proprioceptive receptors and afferent axons were acquired using an Olympus FV1000 confocal microscope with a 60X oil-immersion objective.

### Analysis of Muscle Spindle and GTO Afferents

MS and GTOs were identified by distinguishing morphological characteristics, namely the hallmark annulospiral structure of MS and the highly branched endings of GTOs [5,8–11]. Furthermore, careful attention was paid to whether proprioceptive axons supplying a given MS or GTO reached the ending via the soleus nerve thick branch, thin branch, or both.

Images collected to quantify proprioceptive afferents associated with MS and GTOs were acquired using an Olympus FV300 confocal microscope (20x and 60x objectives). Quantification of the number of proprioceptive axons per spindle was accomplished using Fluoview software (Olympus) together with 3D image visualization software as needed (Imaris 7.7.0, Bitplane). Composite images covering several fields of view were constructed with Photoshop (CS3, Adobe) from maximal intensity projections from confocal image stacks. Using these images, each spindle afferent was reviewed and classified as a primary or secondary ending according to morphological criteria as follows. As every muscle spindle must be supplied by at least one Ia afferent, single afferents solely associated with a MS were classified as primary endings. In spindles with multiple associated afferents, primary endings were identified by classically defined large and small annulospirals indicating extensive wrapping of all three intrafusal muscle fiber types. Secondary endings were distinguished by smaller spirals likely encircling chain fibers as well as by the previously described flower-spray terminal arrangement [8–10]. Group II afferents, however, are also known to contact all three intrafusal muscle fiber types yet the morphology of these endings is overall less extensive than primary endings [10]. In addition to terminal morphology, the spacing of termination points along the MS was also used to classify spindle afferents. Group II endings occupy juxta-equatorial positions at a distance from the equatorial Ia termination zone. Two afferents having Ia morphology and equatorial terminations in close proximity on a given MS were thus classified as multiple primary endings in this study.

Composite images from high magnification (60x objective) confocal scans were also used for intramuscular PSN axon diameter measurements performed by a second reviewer blinded to the receptor innervation and ending of each axon. The blinded reviewer placed markers individually sized to the width of the axon at 5 μm intervals along the length of each axon using Neurolucida (Version 9, MBF). Output data from Neurolucida Explorer was used to calculate the average diameter of all markers placed on an axon, as well as the total length measured (average length measured: 351 ± 238 μm (standard deviation); range: 25–1394 μm; n = 138 axons). Three axons were excluded from analysis because the boundaries between labeled axons and background were obscured.

The diameter data was analyzed with Hartigan’s dip test of unimodality and a logistic regression in R Studio (package = ‘diptest’, Version 0.75–7). Hartigan’s Dip test searched for a statistically significant “dip” in the histogram of diameters to determine whether the data was bimodal [20]. Using a custom script in R, a logistic regression was then applied to estimate the likelihood of correctly classifying a single axon as either a Ia or group II afferent, based on diameter alone. The regression model was also used to determine diameter cut-offs that would provide the best fit with our designation of primary and secondary endings based on morphological criteria. Finally, the diameter data was manually examined by both reviewers and compared with the morphological data. An F-test was conducted in Microsoft Excel to compare the variance of Ib axon diameters to the variance of Ia axon diameters (MS with one axon only, n = 18). A second F-test compared variances between diameters from primaries (Ib and Ia, n = 33) and secondaries (n = 15). Using the results of the F-tests, two separate 2-tailed t-tests were done to compare the same groups of diameters.

## Results

### Assessment of Transgenic Animal Model

A goal of this study was to devise a method for analyzing PSN axons and their specialized endings in a whole muscle preparation. A molecular identifier of PSNs is expression of parvalbumin, a calcium binding protein [21,22]. We therefore employed a Cre/*lox* strategy combining existing transgenic mouse models to produce *PV-Cre; R26-tdT* mice in which all parvalbumin-expressing neurons, primarily PSNs, are labeled with a red fluorescent protein [19,23]. Robust tdTomato signal persisted even after tissue fixation, allowing visualization of PSN axons in whole muscles without signal amplification. Restricted tdTomato expression in muscle PSN was particularly useful for quantification of PSN axons and endings in the soleus of neonatal animals. Parvalbumin is also expressed in extrafusal muscle fibers postnatally [24]. We noted muscle fiber expression already obscured the signal from PSN axons at P0 in muscles with a large proportion of fast-twitch extrafusal fibers, like rectus femoris. The soleus contains a larger percentage of slow-twitch fibers, but in our hands, extrafusal tdTomato signal made it impractical to analyze PSN axons in this muscle after P7. An alternate Cre-driver was thus used to facilitate MS and GTO analysis in older animals. Advillin is expressed by >90% of DRG neurons, including both PSN and non-PSN, but is not expressed in muscle tissue [18]. As with *PV-Cre* controlled tdTomato expression, robust fluorescence was observed even after fixation using the *Adv-Cre* driver line, minus the confounding expression in muscle fibers. Using this Cre-driver, axons of non-PSN (groups III and IV) were also visible in the soleus muscle, although they were not the focus of this study.

**Fig 1:**
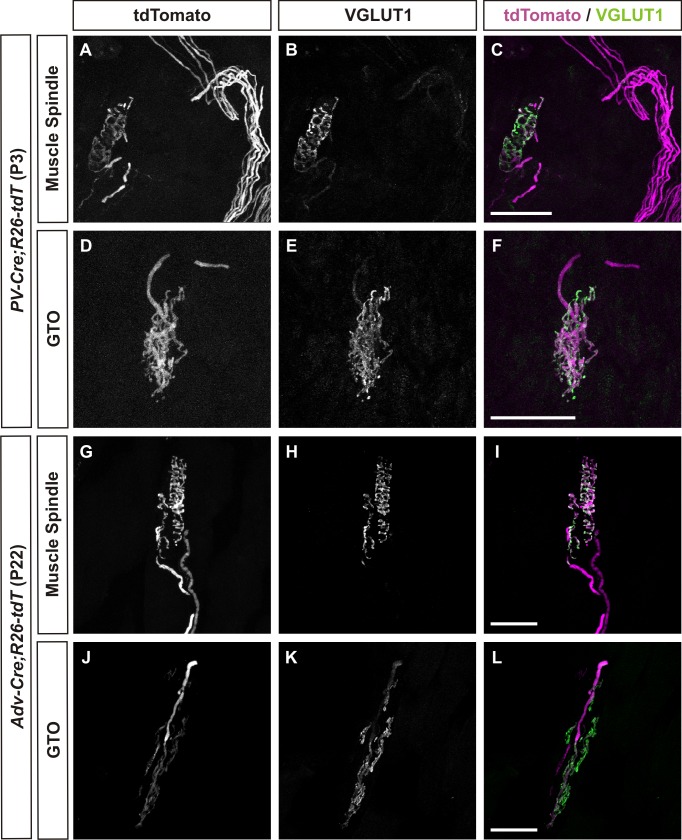
MS and GTO morphology in both neonatal and mature mouse soleus muscles are accurately reported using *PV-Cre; R26-tdT* and *Adv-Cre; R26-tdT* mouse models. Examples of native tdTomato fluorescence in representative muscle spindles (A-C, G-I) and GTOs (D-F, J-L) from each of the mouse models utilized in this study. VGLUT1 expression was largely restricted to the proprioceptive receptor terminals, while tdTomato expression was observed in both proprioceptive receptor terminals and their respective axons. Scale bars represent 50 μm.

To assess the reporter efficiency of our transgenic labeling strategies, we compared immunoreactivity for vesicular glutamate transporter 1 (VGLUT1) to the tdTomato fluorescence of PSN axons and endings in both the *PV-Cre; R26-tdT* and *Adv-Cre; R26-tdT* animals. Representative confocal images in Fig 1 show VGLUT1 expression was largely restricted to the hallmark annulospiral structure of the MS and the branched morphology of the GTO as previously reported [23,25]. Expression of the tdTomato reporter was visualized in proprioceptive endings as well as axons. All VGLUT1 positive MS and GTO structures were innervated by tdTomato axons using either Cre-driver, thus validating these genetic strategies for labeling MS and GTOs in the mouse soleus.

### Quantification of Proprioceptive Receptors

**Fig 2:**
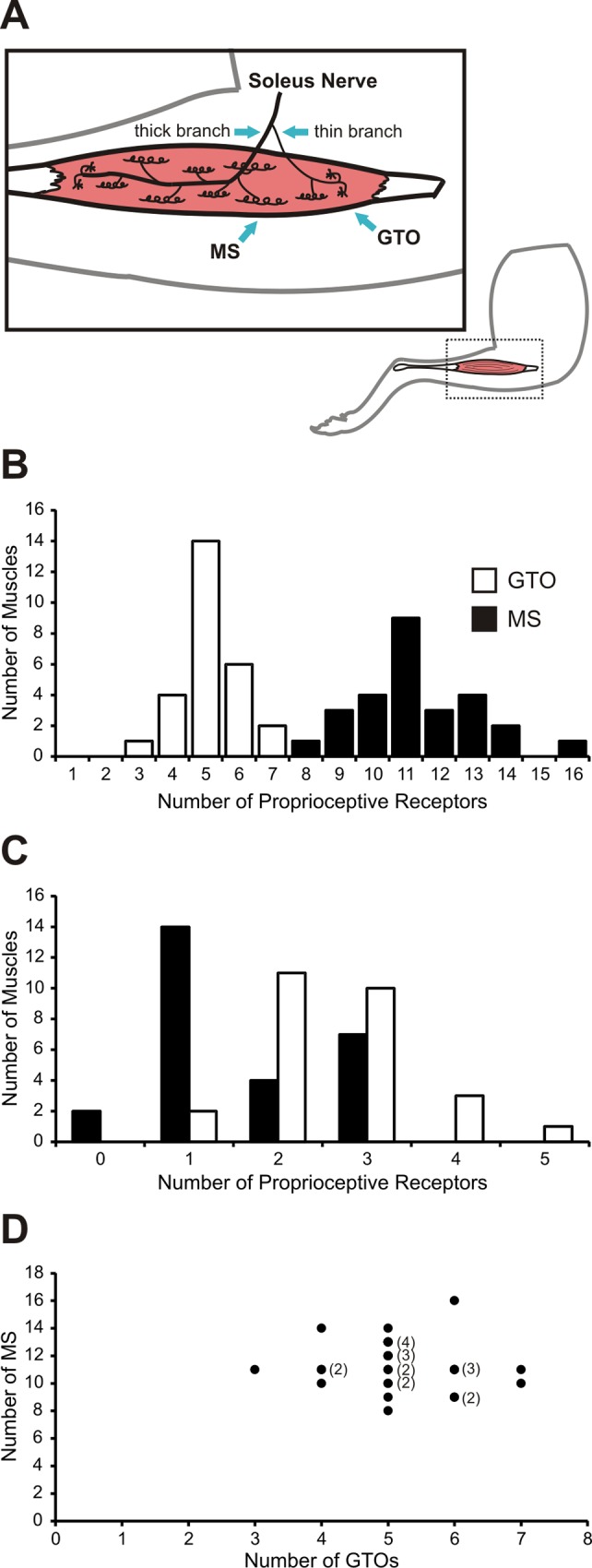
Quantitative analysis of proprioceptive receptors within the mouse soleus muscle. (A) Schematic representation of the extramuscular bifurcation of the soleus nerve into a proximal thin branch and a distal thick branch and the typical distribution of MS and GTOs within the muscle. (B) A survey of 27 muscles from *PV-Cre; R26-tdT* mice and *Adv-Cre; R26-tdT* mice (P3 to P7 and P19 to P20, respectively) found the soleus muscle to contain 11.3 ± 0.4 MS and 5.2 ± 0.2 GTOs (mean ± SEM). (C) Alone, the thin proprioceptive branch of the soleus nerve was found to contain 1.6 ± 0.2 MS and 2.6 ± 0.2 GTOs. (D) Proprioceptive receptor class populations within the mouse soleus varied independently (Spearman correlation *r*_*s*_(25) = -0.1).

Using these genetic tools, we sought to quantify the numbers and distribution of MS and GTOs contained within the intact soleus muscle. Imaging unfixed and lightly compressed soleus muscles from neonatal animals allowed us to rapidly document MS and GTO counts in the entire muscle (Fig 2A). Therefore, the majority of muscles surveyed (23 of 27) were obtained from *PV-Cre; R26-tdT* mice ages P3 to P7. Four muscles were also included from P20 *Adv-Cre; R26-tdT* animals. In total, we found the soleus muscle to contain 11.3 ± 0.4 MS and 5.2 ± 0.2 GTOs (data reported as mean ± SEM; Fig 2B). Our MS and GTO counts for the soleus muscle agreed with previously reported data collected from serial sections (see Discussion).

The soleus muscle is innervated by two small nerves that arise through bifurcation of the common soleus nerve prior to muscle entry (Fig 2A). One branch is consistently thinner than the other and exclusively supplies sensory endings (both MS and GTO) found in the proximal compartment of the muscle. This branch lacks α-motor neuron axons which innervate extrafusal muscle fibers, but does contain γ-motor neuron efferents to MS in the proximal compartment [26]. The thick branch contains all α-motor neuron axons, along with γ-motor neuron and sensory axons that supply the remainder of the muscle. We found on average 1.6 ± 0.2 MS and 2.6 ± 0.2 GTOs innervated by axons from the thin branch (Fig 2C). In 7 of the 27 muscles examined (25.9%) a MS located in the proximal compartment was supplied by PSN axons from both the thick and thin branches of the soleus nerve. In two of the 27 muscles (7.4%) the proximal compartment of the muscle was supplied by two thin branches that diverged prior to entry into the muscle, and each supplied MS and GTOs.

Given the individual variability we observed in the numbers of MS and GTOs contained within the whole soleus nerve, we asked whether above average MS counts were linked to below average GTO counts. Our data suggests that this is not the case (Fig 2D). Animals with above average total MS counts still tended to have GTOs either equal to or above our calculated average number of GTOs. Animals with below average MS counts still maintained the average number of GTOs. Likewise, above average GTO counts did not necessarily indicate fewer MS, and fewer GTOs did not appear to indicate elevated MS. In summary, our analysis showed that numbers of MS and GTOs in the soleus muscle vary independently (Spearman correlation *r*_*s*_(25) = -0.167).

### Analysis of Proprioceptive Sensory Axons

**Fig 3:**
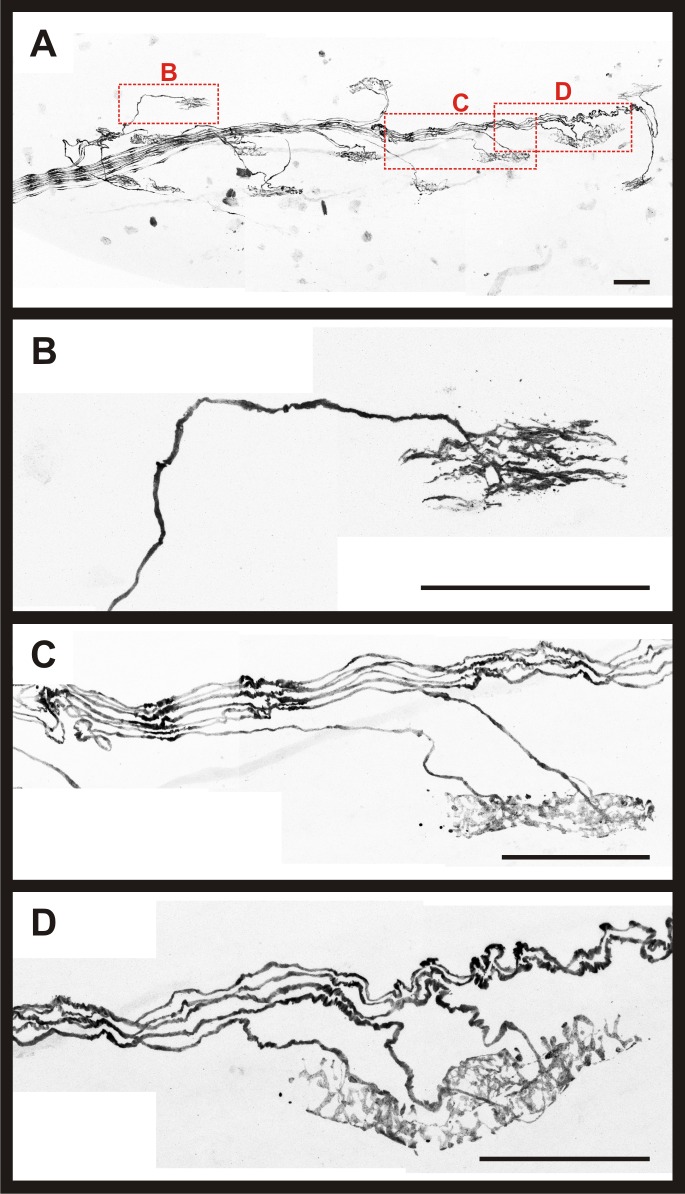
Whole-mount muscle preparation enabled detailed examination of intact proprioceptive innervation of the mouse soleus. (A) Composite 20X confocal image from a P5 female *PV-Cre; R26-tdT* mouse soleus. Representative composite 60X confocal images of a GTO and two MS are shown in (B-D), identified by red boxes. (B) Invariably, GTOs were supplied by a single Ib afferent. (C) Example of a MS supplied by two afferents. (D) Example of a MS supplied by three afferents. Scale bars represent 100 μm.

Another aim of the project was to determine the number and identity of PSNs that project to the soleus muscle. The full complement of MS, GTOs, and associated sensory afferents in a muscle are established before birth in mice [27,28]. Consequently, muscles from both neonatal (*PV-Cre; R26-tdT*) and mature (*Adv-Cre; R26-tdT*) animals were included in this analysis. Intact soleus muscles were again used in these experiments, but muscles were briefly fixed to prevent degradation of the sensory endings. First, the entire muscle was scanned at low magnification to generate composite images to serve as a general map of the proprioceptive innervation (Fig 3A). Second, MS, GTOs, and their associated afferents in each muscle from neonatal and mature animals were scanned at high magnification to obtain detailed images of sensory axons supplying a given proprioceptive receptor (examples in Fig 3B–3D). In these experiments, we analyzed 135 MS and 50 GTOs from 14 muscles (7 *PV-Cre; R26-tdT* and 7 *Adv-Cre; R26-tdT*).

**Fig 4:**
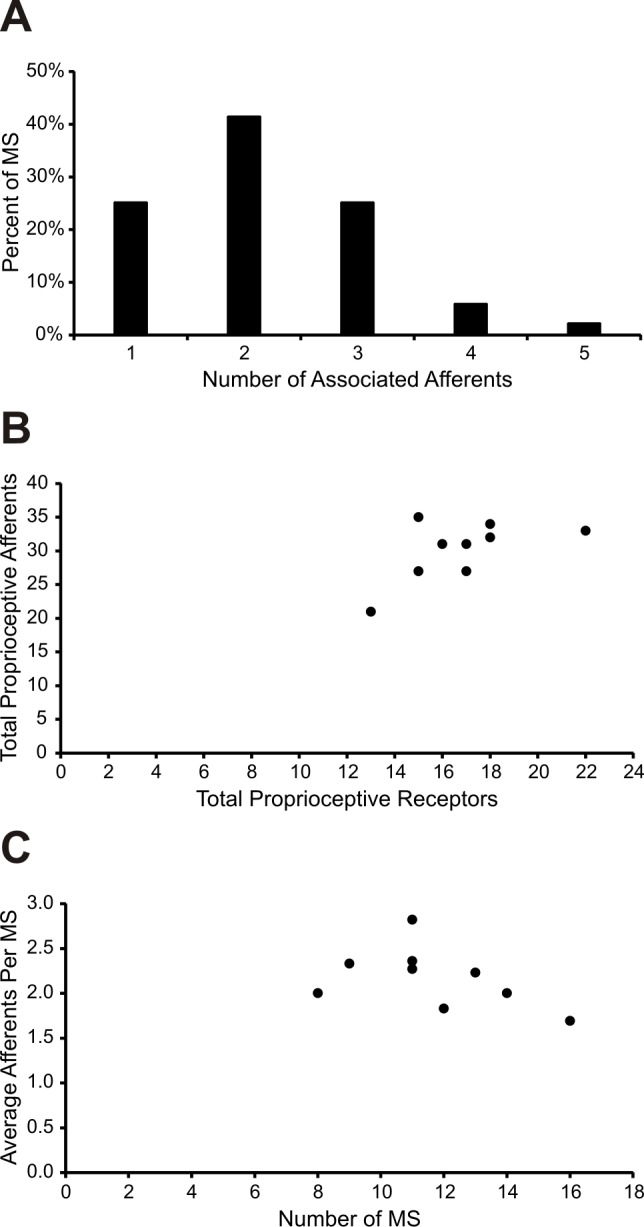
Quantitative analysis of proprioceptive afferents supplying the mouse soleus muscle. (A) The number of proprioceptive afferents supplying a given MS ranged from 1 to 5 (*n* = 135 neonatal and mature MS supplied by 295 proprioceptive afferents in total). Most often, MS were supplied by 2 proprioceptive afferents. (B) Total proprioceptive afferent population per muscle was weakly correlated with total number of receptors (Spearman correlation *r*_*s*_(7) = 0.498). (C) Total number of MS per muscle displayed a modest negative correlation with the number of average afferents per MS (Spearman correlation *r*_*s*_(7) = -0.536).

In every case, confocal imaging confirmed individual GTOs were innervated by a single PSN axon, namely a Ib afferent (n = 50 GTOs; Fig 3B). In only one case, two GTOs were innervated by branches of the same Ib afferent that bifurcated more than 200 μm from the point of entry at the GTOs. The number of PSN axons innervating individual MS varied from 1 to 5 axons. Innervation by two proprioceptive axons was the most common arrangement for sensory supply to MS (41.5% of the 135 MS surveyed; Fig 3C and Fig 4A). In a subset of muscles, all PSN axons were traced back to the main nerve bundles to document the total number of PSN axons projecting via the thin branch as well as the whole soleus nerve. In these experiments, we found the soleus nerve to contain 21 to 36 (30.1 ± 1.5; n = 9 muscles [7 *PV-Cre; R26-tdT* and 2 *Adv-Cre; R26-tdT*]) PSN axons in total. Our analysis further revealed 2 to 7 (4.6 ± 0.5; n = 9 muscles) PSN axons within the thin branch. In all cases, Ib axons were present in the thin branch of the soleus nerve. Spindle afferents, however, were not always present in the thin branch, ranging from 0 to 5 axons (2.1 ± 0.6; n = 9 muscles).

Possessing complete knowledge of the number of MS and GTOs, along with the number of PSN axons innervating these structures in multiple soleus muscles, we investigated the relationship between PSN axons and associated endings in muscles with varying numbers of MS and GTOs. Larger numbers of MS and GTOs were associated with increased numbers of proprioceptive afferents innervating the muscle, although the correlation was weak (Spearman correlation *r*_*s*_(7) = 0.498; Fig 4B). Next we asked if the average number of PSN axons innervating a MS varies with the total number of MS found in individual muscles. We found a modest trend for muscles with more MS to have fewer afferent axons on average (Spearman correlation *r*_*s*_(7) = -0.536; Fig 4C).

**Fig 5:**
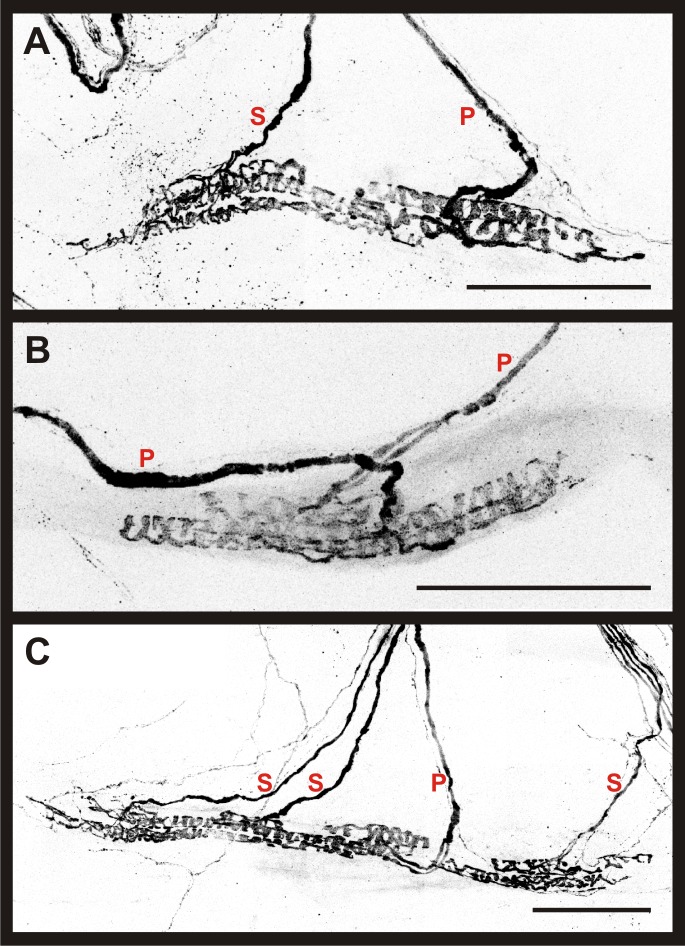
Morphological identification of spindle afferents in the mature mouse soleus. Representative composite 60X confocal images obtained from P19-20 *Adv-Cre; R26-tdT* soleus muscles using the whole-mount muscle preparation. Proprioceptive ending types are labeled in red. P indicates primary afferent ending, and S indicates secondary afferent ending. (A) Example of a MS supplied by two afferents. The primary afferent forms large annulospirals around intrafusal bag fibers, while the secondary afferent terminates primarily in the region of smaller chain fibers. (B) Example of a MS supplied by two primary afferents terminating within close proximity of each other. (C) Example of a MS supplied by four proprioceptive afferents. Scale bars represent 100 μm.

We sought to determine the frequency of primary and secondary MS endings in our dataset using distinctive morphological criteria (see Methods for details). Because overlap of Ia and group II terminations have been observed in neonatal rats [29], only muscles from P20 *Adv-Cre; R26-tdT* animals were included in this analysis. High magnification scans of 60 MS from whole-mount soleus preparations were analyzed to determine the type of each afferent ending. Seventy-five percent of muscle spindles in our dataset fell into one of three categories in terms of sensory innervation. First, the most common arrangement (18/60 MS) was innervation by a single primary afferent. The frequency of the other two main categories, innervation by only two primary afferents (PP; 14/60) or by a primary with two secondary afferents (PSS; 13/60), were similar (see Table 1 and Fig 5 for examples). Among the three MS innervated by 4 or 5 afferents, two received multiple primary endings. No instances of a single, bifurcated axon having more than one ending were noted.

**Table 1:** Shift in proprioceptive afferent configuration observed with increasing number of associated afferents per muscle spindle.

Total Associated Afferents	1	2		3		4		5
Afferent Configuration	P	PP	PS	PPS	PSS	PPSS	PSSS	PPPSS
Number of MS	18	14	8	4	13	1	1	1
Subtotals	18	22		17		2		1

Representative MS (n = 60) from age P19-20 soleus muscles were sorted according to their number of total associated proprioceptive afferents (n = 126 afferents, range = 1 to 5 per MS). Varying configurations of primary (P) and secondary (S) endings identified from morphological criteria were observed when MS had two or more associated afferents. In cases where two afferents supplied a MS, two primary endings (PP) were most frequently observed (14/22 MS). When three afferents supplied a MS, however, the configuration shifted to favor multiple secondary endings (PSS, 13/17 MS).

In addition to characteristic differences in contacts with intrafusal fibers, Ia afferents in cats also have a larger average axonal diameter than group II afferents [10]. We next determined if a similar relationship existed between Ia and group II afferents in the mouse by analyzing proprioceptive axon diameters in mature (P20) mice. A reviewer blinded to receptor ending type measured the intramuscular diameters of all proprioceptive sensory axons supplying 60 MS and 15 GTOs from 7 soleus muscles from P20 *Adv-Cre; R26-tdT* mice. We first analyzed the distribution of diameters for multimodality and found the data to be statistically normal. Next we compared the axon diameters of afferents that must be either group Ia (afferents supplying the 30.0% of MS receiving a single afferent axon) or group Ib afferents innervating GTOs. The variances of the Ib afferent and Ia afferent diameter samples were equal. Therefore equal variance, two-tailed t-tests were performed, indicating that the average diameter of Ib afferents (3.68 ± 0.18 μm; n = 15 axons) was not statistically different from the average diameter of Ia afferents supplying single-axon spindles (3.30 ± 0.11 μm; n = 18 axons; p = 0.07).

**Fig 6:**
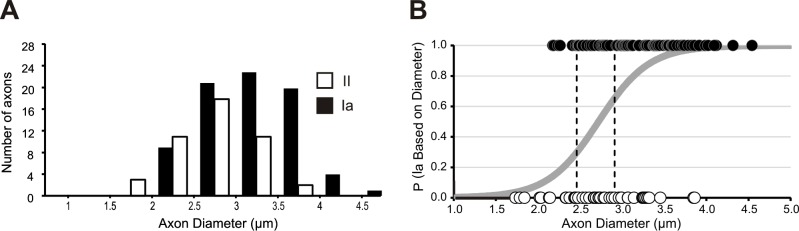
Axon diameter for both morphologically classified spindle afferent subtypes. (A) Histogram of axon diameters for afferents that were classified as primary or secondary endings based on morphological criteria. (B) Logistic regression model used to predict proprioceptive afferent subtypes based on diameter. Black dots represent the diameters of afferents that were classified as primary afferents based on morphologic criteria and therefore they have a 100% probability of being a primary afferent. The open circles illustrate the morphologically classified secondary endings, which have a 0% probability of being a primary ending. The gray line indicates the probability of an afferent being a primary based on diameter. The dotted lines illustrate the cut-off diameters (2.46 and 2.91 μm) for classifying afferents. Axons with diameters left of the first dotted line (diameter < 2.46 μm) were classified as group II afferents, axons with diameters between the dotted lines could not be classified based on diameter alone, and axons with diameters to the right of the second dotted line (diameter > 2.91 μm) were classified as Ia afferents.

To be conservative in the initial assessment of group II afferents, only the smallest axon taken from the subset of MS innervated by three axons was analyzed (n = 17). After unblinding the reviewers, 15 of these 17 axons were shown to have been classified as secondary endings based on morphological criteria. The two axons whose morphological classification and diameter-based prediction differed were removed from the group of presumed group II axons. The average diameter of the remaining group II axons was significantly smaller than the population of presumed primaries (2.49 ± 0.14 μm; n = 15 axons; p < 0.0001). A logistic regression was then applied to all spindle afferents to estimate the likelihood of correctly classifying a single axon as either a Ia or group II axon, based on diameter alone. MS afferents were first classified as primary or secondary endings based on morphological criteria and then these identifications were compared to those predicted by the logistic regression model. The model demonstrated that afferents could be correctly classified by diameter alone 67% of the time (p = 0.0011). To determine diameter cut-offs that would most accurately predict a spindle afferent’s subtype, threshold values were varied. The model had the strongest specificity and sensitivity, 74% and 72% respectively, when the thresholds were set to probabilities greater than 65% and less than 30%. These threshold values corresponded with the diameter measurements of 2.91 μm and 2.46 μm (see Fig 6). Thus, axons with diameters greater than 2.91 μm were at least 65% likely to be Ia afferents. Furthermore, the probability that an ending was a primary afferent increased as the axon diameter increased. For example, any axons with diameters larger than 3.38 μm (32 out of 78 morphologically identified Ia afferents) had a 90% or greater probability of being a Ia afferent based on diameter. Axons with diameters less than 2.46 μm were less than 30% likely to be a Ia, therefore at least 70% likely to be a group II. Predictions of axon type among axons with diameters larger than 2.46 μm and lower than 2.91 μm (19 axons out of 123) were near chance levels (30–65% probabilities) and therefore cannot be classified as Ia or II based on diameter alone. The model revealed an odds ratio of 46, demonstrating that an axon was 46 times more likely to be a Ia afferent with every 1 μm increase in diameter.

The logistic regression was conducted because Hartigan’s dip test determined that the combined population of all spindle afferents was statistically normal even though previous literature has reported that axon diameters follow bimodal distributions in the cat [30]. Conversely, the logistic regression demonstrated that axon diameter could classify axons into two distinct groups, with a high degree of accuracy. We found the diameters of each afferent subtype spanned almost the entire range of diameters; the range of primary endings was 2.16–4.54 μm and the range of secondary endings was 1.73–3.86 μm. In addition to these large ranges, 70% of the axon diameters for both subtypes fell within the range of 2.75–3.75 μm. This high degree of overlap created a normal distribution despite the statistically significant differences between groups.

Both morphological and axon diameter approaches contribute to our understanding of the afferent types innervating individual MS. Applying this information to the total complement of MS (11.3) and GTOs (5.2) found in the soleus, it was possible to estimate the total number of Ia, Ib, group II axons that innervate the soleus. Given GTOs were always observed to be innervated by a single Ib afferent, approximately 5 Ib afferents project to the soleus muscle. From the subset of MS analyzed in Table 1, 81 of the 126 MS axons analyzed were found to make primary endings, while the minority of afferents (45 of 126) formed contacts consistent with secondary endings. The combination of these ratios suggests a MS on average is innervated by 2.1 afferents and that primary afferents outnumber group II afferents approximately 2:1 (1.35 Ia: 0.75 group II per spindle). Thus, for an average soleus muscle (11.3 MS), 15.3 Ia and 8.5 group II axons would make up the afferent supply to MS. The combined total of 23.8 MS afferents and 5 Ib afferents supplying GTOs (28.8 PSN axons total) is in agreement with the average number of PSN axons in whole soleus determined in our study (30.2 ± 1.5; n = 9 muscles).

## Discussion

In this study we used a transgenic reporter system to analyze the sensory innervation of MS and GTOs using an intact mouse soleus muscle preparation. Robust fluorescence in PSN axons permitted visualization of afferent pathways and terminations within a muscle with a minimum of specimen preparation and no tissue sectioning, representing a substantial gain in efficiency compared with serial section or teased preparation techniques. This whole-mount muscle approach enabled quantification of proprioceptive receptors and their respective afferents as well as comparison of spindle sensory ending classifications obtained via morphological identification and intramuscular axonal diameter measurement.

When controlled by either *PV-Cre* or *Adv-Cre* driver lines, the *R26-tdTomato* reporter line generated strong fluorescence in sensory axons that required no amplification following brief fixation. Use of the *PV-Cre* was desirable because among muscle sensory afferents, parvalbumin expression is limited to only proprioceptive axons [19,23]. During postnatal maturation, however, expression of parvalbumin in extrafusal muscle fibers [24] leads to widespread tdTomato signal in the muscle, rendering proprioceptive axons indistinguishable from the surrounding fibers. Driving tdTomato expression with *Adv-Cre* eliminated expression in muscle fibers, and revealed a dense network of sensory axons and endings throughout the muscle. In addition to PSN axons, smaller caliber axons (likely group III and IV) were widely distributed in the muscle [31]. These axons were never found within MS capsules and did not interact with Ib afferent endings at GTOs. Recently developed genetic tools offer the possibility of even greater selectivity in reporter expression using intersectional strategies that employ two distinct recombinase proteins (Cre and Dre, for example) to restrict expression to only a subset of cell types that would be labeled using only a single recombinase [32]. Such a strategy could be utilized in this system to eliminate tdTomato (or other reporter) expression in extrafusal muscle fibers, while still limiting neuronal expression to only proprioceptive afferents in muscles.

MS in the mouse soleus have been quantified in previous reports, usually as part of a larger research question with fewer replicates than the present study. Our reported average of 11.3 ± 0.4 MS occupies the middle of the range of numbers from 10 to 12 MS reported elsewhere [17,33–38]. GTO numbers in mouse soleus have been less frequently quantified, but again numbers reported here (5.2 ± 0.2) agree with published reports [36,39]. Thus, the whole-mount muscle preparation used in these experiments allowed not only for rapid quantification of proprioceptive receptors and larger sample sizes, but also yielded equally accurate measures compared with more labor-intensive techniques.

Analysis of MS and GTOs using an intact muscle allowed us to readily quantify endings from a relatively large sample of muscles, revealing a wide range of natural variability in both the number of MS, GTOs, and the PSN axons supplying these structures. Could the natural range of proprioceptive endings, two-fold for MS (8 to 16) and GTOs (3 to 7) influence the proprioceptive sensitivity of individual animals? Total loss of muscle spindles has an obvious impact on locomotor behavior, but intermediate reductions in spindle number have less obvious effects. Muscle spindles do not form in mice lacking neurotrophin-3 (NT-3), a trophic factor necessary during embryogenesis for PSN survival, and approximately half the normal complement of muscle spindles are found in mice heterozygous for the mutation [39]. Nevertheless, no obvious behavioral phenotypes are observed in *NT-3* heterozygous animals [39]. This suggests sufficient feedback can be derived from a limited set of muscle spindles and implies spinal circuits can adapt to varying levels of feedback present during development. Conversely, overexpression of NT-3 in skeletal muscle increases the number of PSNs in the DRG and can result in increased numbers of spindles in limb muscles. Less favorable adaptation of the nervous system occurs in this condition, however, as evidenced by measurable disturbances in gait and coordination in these animals [40,41]. Nevertheless, the contribution of increased numbers of spindles to such phenotypes is speculative as the specificity of afferent to motor neuron connections in the spinal cord is disrupted in these animals and is likely a major contributor to coordination deficits [42].

The uncorrelated variability in MS and GTO numbers in individual muscles suggests no consistent ratio of MS and GTOs is maintained in the soleus. While the mechanisms involved in MS and GTO induction remain unclear, alterations in gene expression can favor development of one receptor type at the expense of the other. In mice lacking Er81, an ETS-family transcription factor expressed by all PSNs and also induced in intrafusal fibers during development, the number of MS in the soleus increased while the number of GTOs decreased [43,36]. Dual expression of Er81 in both the PSNs and their intrafusal muscle fiber targets makes it difficult to parse out the specific role of Er81 in sensory neurons. However, the fact that Er81 selectively increases the number of MS in the soleus supports our findings that MS and GTO numbers can vary independently and further, suggests that transcription factors like Er81 may play a role in the variability of MS and GTO numbers.

We found muscle spindle primary (Ia) and secondary (II) afferent populations of mature mice are significantly different in terms of intramuscular diameter, but the distributions share substantial territory. Overlap in the diameters of group Ia and II axons is also observed in the cat, with up to 75% of soleus group II axons being found within the range of Ia afferent axon diameters [10]. The degree of overlap in our data is even higher with only 13% (6/45) of group II axons having axon diameters smaller than any muscle spindle primaries. Evidence of overlap is also observed at the upper end of the range, with only 6% (5/81) of Ia afferents extending beyond the group II range. More separation between the diameters of spindle afferent subtypes is evident in cats with ~18% of MS primaries being larger than secondaries [10]. In our study, axon diameters were measured only from P20 mice, a stage when MS have morphologically matured. Further divergence in axon diameters may occur in older animals. Nevertheless, a study with rats also found the distribution of conduction velocities, a physiological parameter related to axon diameter, to be not as distinctly bimodal as it is in cats [12].

To date, the most detailed anatomical analysis of the afferents supplying MS in rodents has come from rats, and this data suggested spindles in the soleus muscle frequently receive multiple primary afferent endings (~65% of spindles), a finding in agreement with our results, but which contrasts with the predominant single primary afferent pattern observed in the classic feline animal model [16]. The prevalence of multiple primary spindle afferents varies between muscles. Nearly all spindles (94%) in the rat masseter muscle, but only 45% in the lumbrical muscles, are supplied by multiple primaries [16]. It has been suggested that the more temporally compressed developmental sequence of spindle initiation and innervation in rodents compared with cats may lead to increased prevalence of multiple primary afferents at a spindle [16]. Physiological characterization of MS afferents in the rat masseter muscle indicated most afferents have intermediate responses that are not clearly classifiable as primary or secondary in nature, suggesting MS supplied by multiple Ia afferents may not encode muscle stretch in the same way as a single Ia afferent supplied MS [44].

In summary, genetic reporter lines were used in this study to comprehensively analyze the proprioceptive components of the mouse soleus muscle. These mouse lines enabled us to examine a whole soleus muscle, accelerating the process of quantifying MS and GTO counts. Receptor counts demonstrated that there are approximately twice as many MS as GTOs on average, and that the number of each receptor type varies independently from the other in the soleus. Morphological analysis of PSN axons revealed that most muscle spindles are innervated by two or three axons, with variable numbers of primary and secondary endings. Overall, morphological classifications of spindle afferent subtypes matched classifications by axon diameter. However, due to the substantial degree of overlap in the diameters of Ia and II axons, over a third of afferents could not accurately be classified by their diameters. Combined with findings in the rat, the overlapping ranges of Ia and II axon diameters in this study suggest that in the rodent, conduction velocity and axon diameter only distinguish afferent subtypes at the farthest extremes of data distributions, and therefore reinforce the need for careful morphological characterization or more robust electrophysiological techniques to accurately classify primary and secondary endings [12]. Taken together, these results demonstrate the utility of genetic reporter lines to facilitate rapid and accurate analysis of proprioceptive receptors in whole-mount muscle preparations. Future studies could exploit advantages of transgenic mouse models to investigate potential differences in stimulus encoding between MS supplied by single or multiple primary afferents.
